# Development of a core outcome set for clinical trials in childhood asthma: a survey of clinicians, parents, and young people

**DOI:** 10.1186/1745-6215-13-103

**Published:** 2012-07-02

**Authors:** Ian P Sinha, Ruairi Gallagher, Paula R Williamson, Rosalind L Smyth

**Affiliations:** 1 Institute of Child Health, University of Liverpool, Alder Hey Children’s Hospital, Alder Road, Liverpool, L12 2AP, UK; 2 Centre for Medical Statistics and Health Evaluation, University of Liverpool, Brownlow Hill, Liverpool, L69 3GS, UK

**Keywords:** Asthma, Core outcome set, Delphi, Children, Paediatrics

## Abstract

**Background:**

In clinical trials in childhood asthma, outcomes reflecting short-term disease activity are frequently measured, whilst functional status, quality of life (QoL), and long-term treatment effects are rarely assessed. There is also non-uniformity across studies in the selection and measurement of outcomes within these domains. The development of a core outcome set has the potential to reduce heterogeneity between trials, lead to research that is more likely to have measured relevant outcomes, and enhance the value of evidence synthesis by reducing the risk of outcome reporting bias and ensuring that all trials contribute usable information.

**Methods:**

Paediatricians and specialist nurses, identified through the British Paediatric Respiratory Society, completed a two-round Delphi survey. Separate cohorts of parents of children younger than 18 years, recruited in clinics, participated in each round. Young people with asthma, aged at least 13 years, participated in the first round. Outcomes were identified separately for preschool and school-aged children.

We identified outcomes considered important in routine clinical assessment by clinicians and parents/young people. In round 1, 46 clinicians suggested outcomes they considered important when deciding whether to adjust a child’s asthma therapy regime, and 49 parents/young people were asked, using open questions, how they judged whether their child’s (for young people, their own) asthma therapy was appropriate. Two researchers independently classified responses into appropriate, corresponding outcomes.

In round 2, 43 clinicians and 50 parents scored, from 0–4, the importance of each outcome suggested by at least 10 % of round 1 responders and selected the three most important.

**Results:**

The most important outcomes, when making shared decisions about regular therapies for school-aged and preschool children with asthma, were daytime and nocturnal symptoms, exacerbations, QoL, and mortality. Results from parents and clinicians were generally concordant, but parents placed more emphasis on long-term treatment effects.

**Conclusions:**

We have developed a methodology to identify outcomes of most relevance to clinicians, parents, and young people when evaluating regularly administered therapies for asthma. Daytime and nocturnal symptoms, exacerbations, QoL, and mortality are particularly important outcomes that should be measured and reported in all clinical trials of regular therapies for children with asthma.

## Background

To inform clinical practice, clinical trials that aim to determine benefits and risks of treatments should measure outcomes that are important to patients, and useful to clinicians and policymakers. It can be difficult, however, for trialists to know which outcomes are most important for a given condition. Chronic illnesses can affect the lives of patients and families in a variety of ways, each of which can potentially be improved by an intervention and could therefore be measured as an outcome.

In a systematic review, of studies that aimed to determine which outcomes to measure in clinical trials in children [1], we found only one study relating to asthma [2]. In this study, 14 clinicians and researchers indicated, by questionnaire, which outcomes they felt were most appropriate for a variety of clinical, public health, and research settings related to asthma. More recently, the American Thoracic Society (ATS) and European Respiratory Society (ERS) held workshops, attended by 24 clinical researchers, with the aim of recommending which outcomes to select in clinical trials of regular therapies for asthma and how these could be measured in a standardised manner [3]. These recommendations relate to adults and adolescents, but the authors suggest that, with some special considerations, the outcomes could also be relevant for children older than 6 years.

In another systematic review, we aimed to identify which outcomes and domains were measured in randomised controlled trials (RCTs) of inhaled corticosteroids for children with asthma [4]. We found that studies almost always measure clinical and physiological markers of short-term disease activity, but quality of life (QoL), functional status, and long-term outcomes are rarely assessed. We also noted marked inconsistency between studies in the way that particular outcomes are measured and reported, and non-uniformity in the definitions of clinical events, especially exacerbations, used as endpoints.

We recommended that one solution to these problems would be to agree on a minimum set of core outcomes that should be measured and reported in all RCTs that assess the effectiveness of treatments given to prevent symptoms and complications in children with asthma. Core outcome sets increase the likelihood that important outcomes will be measured, improve evidence synthesis by reducing heterogeneity between studies, and reduce outcome-reporting bias [5]. Such core outcome sets have been implemented in other conditions, notably in the field of rheumatology. The OMERACT (Outcome Measures in Rheumatology) collaboration has designed core outcome sets for various conditions by reaching consensus amongst clinicians and researchers about which outcomes to measure [6], and more recently by asking patients which outcomes they feel are most important [7]. The COMET (Core Outcome Measures in Effectiveness Trials) Initiative brings together researchers interested in the development and application of core outcome sets [8]. Data on over 120 published or on-going studies related to core outcome set development have been entered into the COMET repository (http://www.comet-initiative.org). The outcomes measured in clinical trials, in order to evaluate whether the benefits of a treatment outweigh its harms, should be aligned to the assessments that are used in clinical practice to decide whether a treatment regime is satisfactory. Core outcome sets do not need to comprise an extensive list of outcomes, but rather a few particularly important ones that reflect the ways in which patients, families, and clinicians assess whether a treatment regime is satisfactory, and make shared decisions about whether to continue or modify it. There is little guidance, however, for researchers who wish to involve these groups in the development of a core outcome set.

The aim of this study, therefore, was to develop and pilot a method by which to identify outcomes of particular relevance when evaluating the effects of regular therapies for chronic childhood asthma, from the perspective of clinicians involved in the out-patient management of children with asthma, parents of children younger than 18 years, and young people aged between 13 and 18 years. In previous attempts to develop core outcomes sets, participants have generally considered outcomes that had already been measured in clinical trials [8,9]. This approach may overlook important outcomes if they have not previously been routinely measured and perpetuate the inclusion of others, which may be less relevant. We therefore took an empirical approach of focusing on aspects of a child’s symptoms or history that would lead to a conclusion by a clinician, parent, or young person that treatment was either adequate or not.

## Methods

### Ethics statement

We asked the advice of the National Research Ethics Service (NRES) about whether this study required ethical review by an NHS Research Ethics Committee, and they advised that this should be considered as service evaluation and development. No identifiable details about patients or families were collected. To acknowledge the clinicians who participated in the study, we have listed them, having obtained consent individually to do so, in Additional file 1.

This study was conducted, using questionnaires, in two phases (1 and 2). The purpose of phase 1 was to identify a long list of potential outcomes, and phase 2 was designed to identify which of these were most important. Participants were asked to consider outcomes for evaluating therapies taken regularly rather than for acute exacerbations. Outcomes for preschool (younger than 5 years) and school-aged (at least 5 but not yet 18 years) children were considered separately. This was because not all outcomes can be measured in younger children, and different outcomes may have varying relevance at different ages. For example, physiological tests of lung function are rarely measured in preschool children because of technical difficulties, and functional status may be assessed in different ways because the pattern of normal daily activities changes when children start school.

To ascertain the views of clinicians, all members of the British Paediatric Respiratory Society (BPRS) and a network of asthma nurses were invited to participate in a two-round, web-based, anonymised Delphi survey. The Delphi technique, which has been used to develop other core outcome sets [9], is a structured method for reaching consensus, in which participants complete sequential rounds of questionnaires, with the results of each questionnaire informing the composition of the next. The BPRS comprises medical and non-medical professionals within the UK who care for children with respiratory problems. To be eligible to participate, we invited clinicians and nurses who had current experience of managing children with asthma, but not necessarily experience of designing or conducting clinical trials. The selection of participants on the basis of relevant clinical experience, rather than research expertise, differs from the approach generally taken when core outcome sets have been developed.

Parents were invited to complete paper-based surveys in asthma clinics in Alder Hey Children’s Hospital (AHCH), a large paediatric hospital in the North of England, where patients are referred from primary and secondary care. Parents of all children younger than 18 years who were prescribed regular preventer therapy for asthma and did not have respiratory co-morbidities were eligible. Young people aged 13 to 18 who attended these clinics were also invited, because we anticipated that they might have different goals for their asthma treatment. The lower age limit was based on clinical experience that teenagers are generally more able to discuss their asthma than younger children. When participants were approached in the outpatient department, one reviewer (IS) explained verbally what was meant by the terms clinical trials and outcomes, and why we were conducting the study. We elected to use questionnaires rather than focus groups to minimise the burden on participants and to enable the involvement of a larger sample. We also felt that participants’ opinions did not require in-depth analysis, which would have been best answered using qualitative research techniques.

### Phase 1

Phase 1 comprised the first round of the Delphi survey of clinicians, and a survey of parents and young people. Open questions were asked in order to identify a long list of outcomes that could be relevant to clinic consultations. In a pilot phase, we asked parents and young people with asthma, and a group of young people with experience of reading clinical trial information leaflets whether the questions were easy to understand and appropriate; the questions were then refined accordingly with extensive input from parents and young people. The questions included in the questionnaires are listed in Table 1.

**Table 1:** Questions asked in the phase 1 questionnaires distributed to clinicians, parents, and young people

Clinician questionnaire	“When you see children with asthma in clinic, you make an assessment as to whether their treatment is working. Please list up to five beneficial or harmful outcomes of treatment that you find clinically most important in school-aged/preschool children. These factors should be things that you consider, when deciding whether to recommend continuing on current treatment or altering a child’s regular asthma therapy regime”
Parents (young peoples’) questionnaire	“Over the last 12 months, have you generally felt that the regular preventer treatment that your child (you) takes has kept their asthma under control? Yes/No. If you ticked YES, please tell us what aspects of your child’s (your) asthma, or their daily life, have made you feel happy that they are on the correct regular medication. If you ticked NO, please leave this question blank”
	“Over the last 12 months, have there been times when you felt that your child’s (your) regular preventer treatment should be increased or changed, because their (your) asthma was not under control? Yes/No. If you ticked YES, please tell us the reasons why you were not satisfied with the regular preventer treatment that they (you) were taking? If you ticked NO, please leave this space blank”
	“Does anything worry you about the fact that your child (you) has asthma? Yes/No. If you ticked YES, please tell us the worries you have about the fact your child (you) has asthma. If you ticked NO, please leave this space blank”
	“Does anything worry you about the regular preventer treatment that your child (you) takes for their asthma? Yes/No. If you ticked YES, please tell us what worries you have about the treatment your child takes for their asthma. Please be as specific as you can. If you ticked NO, please leave this question blank”

IS interpreted each response from clinicians, parents, and young people, and decided which outcome of treatment was being described. The broad framework for classifying responses as outcomes was based on the domains, subdomains, and outcomes identified in the systematic review described earlier [4]. This comprised the following six categories:

· Short-term disease activity: Symptoms; relief inhaler use; exacerbations; lung function; overall asthma control

· Physical consequences of disease: death; progression of asthma into later childhood or adulthood

· Functional status: ability to exercise or play sport; activities of daily living; school attendance

· Family outcomes and Quality of Life: overall QoL; emotional well-being; family outcomes

· Adverse effects of therapy: short-term adverse effects; long-term adverse effects

· Health resource utilisation

Four reviewers (IS, RG, PRW, RLS) discussed whether each of the responses that did not fit into this classification should constitute a ‘new’ outcome.

In order to identify responses that were open to interpretation and to make categorisation of responses more accurate, reviewer 2 (RG) independently categorised the first 36 (72 %) questionnaires received from parents and young people, and reviewer 3 (PRW) independently analysed a randomly selected sample of 9/46 (20 %) of the questionnaires completed by clinicians. Disagreements were discussed among all four reviewers. Further review of the responses from parents and young people was not deemed necessary because interpretation of responses became easier and agreement became greater as the study progressed. The responses from clinicians were easier to interpret and categorise, and agreement among reviewers was excellent, so further review of these questionnaires was not deemed necessary.

To enable each group of participants, regardless of its size, to have equal opportunity to suggest outcomes for phase 2, those outcomes suggested by at least 10 % of young people and/or parents and/or clinicians were carried forward to the next phase. By censoring in this way, we reduced the number of outcomes listed on the phase 2 questionnaire, without overlooking outcomes of potentially genuine importance. The reviewers discussed the individual outcomes that had not been suggested by sufficient numbers of participants, but were measured in at least 10 % of RCTs identified in the systematic review described earlier [4]. If nearly 10 % of both clinicians and parents suggested the outcome, it was carried forward to the next phase, because we felt that, if we had a larger sample size, the outcome may have been suggested by sufficient numbers of participants.

**Figure 1:**
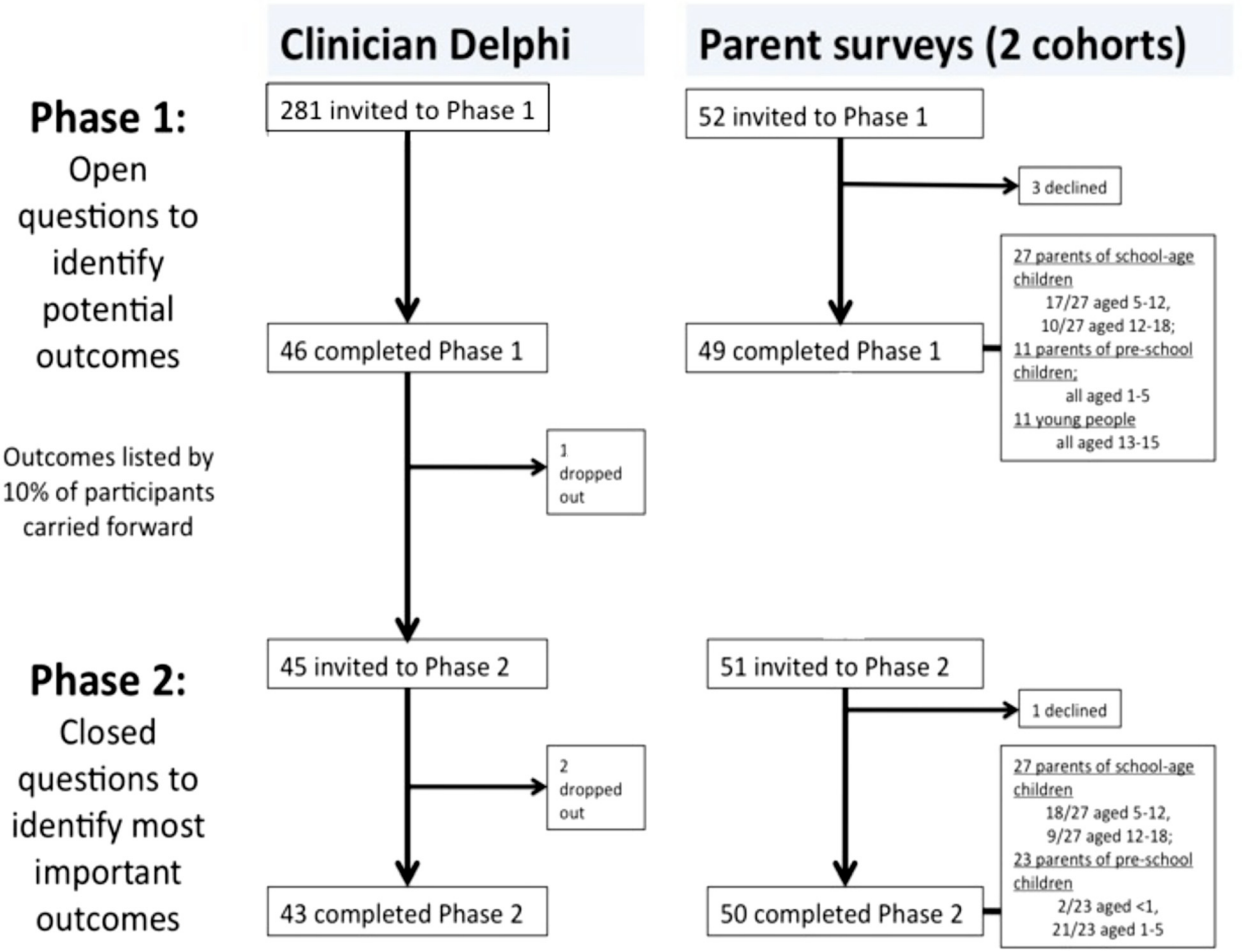
Study flowchart showing participants in each phase of the study.

The study flowchart is shown in Figure 1. At the start of the study, 260 members of the BPRS and 21 specialist asthma nurses were invited to participate. Of the 46 respondents, who came from both district general hospitals and tertiary respiratory centres, 38 were paediatricians (of whom 16 were specialist respiratory paediatricians, 16 were general paediatricians, and 6 were clinical academics), and 8 were respiratory nurses. The participants are listed in Additional file 1. In total, 38 parents (of whom 27 attended with school-aged children and 11 attended with preschool children) and 11 young people, ranging from 13 to 15 years, participated in phase 1.

### Phase 2

Only the clinicians who participated in phase 1 were invited to complete the phase 2 questionnaire. We assumed that if clinicians did not respond to phase 1, they would be unwilling or unable to participate in phase 2, so we took the pragmatic decision not to contact them again at this stage. Parents attending asthma clinics at AHCH were invited to participate, employing the same eligibility criteria as those used in phase 1. We felt confident in running a Delphi process with clinicians, but felt that questionnaires to parents should be delivered in person, so that the questions could be explained. The advantages of doing so would be that the questions would be better understood, and the response rate to the phase 2 questionnaire would be higher. We decided to use a new cohort for the second round of the study so that parents involved in phase 1 would not need to be contacted outside their clinic appointment. We felt that two cohorts would be sufficiently similar to enable this approach because phases 1 and 2 were conducted in identical clinics, so we assumed that family demographics and the severity of the children’s asthma, were comparable across the two phases of the study.

When parents were invited to participate, the recruiting researcher (IS) explained the purpose of the study and the use of the term ‘outcome’ verbally. To increase numbers, parents of preschool children were recruited at a local district general hospital, whose paediatric respiratory department treats children with asthma with broadly similar characteristics to those seen at AHCH. As it was unclear whether young people would be able to answer the questionnaire in phase 2, they were not included. Unlike questions asked in phase 1, those questions asked in phase 2 did not reflect actual discussions that take place with young people in clinic consultations. We felt that in order to involve young people in phase 2, the questionnaires would need to be rigorously validated in this population, and this would be outside the scope of the study. In another attempt to increase the size of the sample, the phase 2 questionnaire was distributed by Asthma UK, the largest UK-based asthma charity, to parents of children with asthma, who had previously expressed an interest in being involved in clinical research.

The questionnaires distributed to parents and clinicians in phase 2 are included in Additional file 2. To identify the relative importance of each outcome, participants were asked the following question: “Regular treatments for children can have a variety of beneficial effects, each of which could be measured as an outcome in clinical trials. Please score how important each of the following outcomes are on a scale of 0–4”. They were also asked to pick the three outcomes they felt were most important. In order to ensure that important outcomes were not missed, participants were asked to suggest any unlisted outcomes that they would have selected in their top 3.

The results from clinicians and parents were analysed separately, and those of parents of preschool children were analysed separately from those of parents of school-aged children. Each outcome was ranked in terms of the proportion of participants who selected it in their top 3.

Phase 2 was completed by 43 clinicians and 50 parents (of whom 27 attended with school-aged children and 23 attended with preschool children). In spite of several reminders, only 13/118 (11 %) responses were received from Asthma UK. As a result, these data have not been included in our analyses.

## Results

### Identification of outcomes in phase 1

The results of phase 1 are shown in Additional file 3. Participants suggested a total of 29 outcomes, of which symptoms, and their effect on ability to exercise and lead a normal life, exacerbations, and adverse effects of therapy were most frequently suggested. One outcome (QoL) was carried forward to the phase 2 questionnaire for preschool children despite not being suggested by 10 % of any group of participants, because it was suggested by 9 % of both parents and clinicians.

There was good agreement among reviewers with regards to how most responses should be categorised, according to the framework of outcomes described earlier. Responses relating to long-term outcomes, however, were more difficult to categorise, because it was often unclear whether participants referred to future risk of asthma, other respiratory illness, general health problems, or effects on activities of daily living, and we were unsure whether these related to later childhood or adulthood. We felt it would be appropriate to combine these responses into the outcome ‘health-related problems when older’. A summary of the agreement among reviewers is shown in Additional file 4.

### Ranking of outcomes in phase 2

All the outcomes listed in the phase 2 questionnaire were considered important. By asking participants to select their top 3, we were able to identify those of particular importance.

The results for each outcome listed in the phase 2 questionnaire relating to preschool children are shown in Table 2. The most important outcomes for clinicians and parents were symptoms, especially those arising at nighttime, exacerbations, QoL, mortality, and hospital admission. The results for each outcome listed in the phase 2 questionnaire relating to school-aged children are shown in Table 3. The most important outcomes in this age group were symptoms, exacerbations, QoL, ability to perform normal activities, and mortality.

**Table 2:** Preschool children: the importance of each outcome listed in the phase 2 questionnaire, as scored by clinicians and parents

	**Clinicians (*n*** **= 43)**			**Parents (*n*** **= 23)**		
**Outcome**	**Clinician rank**	**Median (IQR)**	**Number (%) scoring outcome in top 3**	**Parent rank**	**Median (IQR)**	**Number (%) scoring outcome in top 3**
**Nocturnal symptoms**	1	4 (3, 4)	19 (44)	1=	4 (4, 4)	10 (43)
**Exacerbations**	2	4 (3, 4)	15 (35)	3	4 (4, 4)	7 (30)
**Quality of life**	3	3 (3, 4)	13 (30)	4	4 (3, 4)	5 (22)
**Daytime symptoms**	4	4 (3, 4)	11 (26)	10	4 (3, 4)	3 (13)
**Death**	5	4 (2, 4)	10 (23)	1=	4 (4, 4)	10 (43)
**Hospital admission**	6	4 (3, 4)	9 (21)	7	4 (4, 4)	4 (17)
**Parent/child global assessment of control**	7	4 (3, 4)	9 (21)	11	4 (3, 4)	3 (13)
**Impact of asthma on the family**	8	4 (3, 4)	8 (19)	16	4 (3, 4)	1 (4)
**Use of reliever**	9	3 (3, 4)	8 (19)	8	4 (3, 4)	4 (17)
**Normal activities**	10	4 (3, 4)	5 (12)	14	4 (3, 4)	2 (9)
**Long-term AE**	11	4 (3, 4)	7 (16)	13	4 (4, 4)	2 (9)
**School attendance**	12	3 (3, 4)	5 (12)	6	4 (3, 4)	5 (22)
**Activity or exercise**	13	3 (3, 4)	4 (9)	12	3 (2, 4)	3 (13)
**GP/A + E attendance**	14	4 (3, 4)	4 (9)	15	4 (3, 4)	2 (9)
**Growth**	15	3 (3, 4)	3 (7)	9	4 (3, 4)	3 (13)
**Health-related problems when older**	16	3 (2, 4)	0	5	4 (4, 4)	5 (22)
**Short-term AE**	17	3 (2, 3)	0	17	3 (3, 4)	0

**Table 3:** School-age children: The importance of each outcome listed in the phase 2 questionnaire, as scored by clinicians and parents

	**Clinicians (*n*** **= 43)**			**Parents (*n*** **= 27)**		
**Outcome**	**Rank (*n*/17)**	**Median (IQR)**	**Number (%) scoring outcome in top 3**	**Rank**	**Median (IQR)**	**Number (%) scoring outcome in top 3**
**Quality of Life**	1	4 (3, 4)	20 (47)	2	4 (4, 4)	9 (33)
**Exacerbations**	2	4 (4, 4)	18 (42)	3	4 (4, 4)	9 (33)
**Nocturnal symptoms**	3	4 (4, 4)	16 (37)	4	4 (4, 4)	8 (30)
**Normal activities**	4	4 (4, 4)	12 (28)	8=	4 (4, 4)	4 (15)
**Daytime symptoms**	5	4 (4, 4)	11 (26)	6	4 (4, 4)	6 (22)
**Death**	6	4 (2, 4)	11 (26)	1	4 (4, 4)	14 (52)
**Use of reliever inhaler**	7	4 (3, 4)	9 (21)	10	4 (3, 4)	3 (11)
**Hospital admission**	8	4 (3, 4)	8 (19)	12	4 (4, 4)	2 (7)
**Activity or exercise**	9	4 (3, 4)	7 (16)	5	4 (3, 4)	7 (26)
**Long-term adverse effect**	10	4 (4, 4)	7 (16)	8=	4 (4.4)	4 (15)
**School attendance**	11	4 (3, 4)	7 (16)	11	4 (4, 4)	2 (7)
**Parent/child global assessment of control**	12	4 (3, 4)	7 (16)	14	4 (3, 4)	2 (7)
**Lung function tests**	13	3 (2, 3)	5 (12)	15	4 (3, 4)	1 (4)
**GP/A + E attendance**	14	4 (3, 4)	3 (7)	16	4 (3, 4)	0
**Growth**	15	4 (3, 4)	2 (5)	13	4 (3, 4)	2 (7)
**Health-related problems when older**	16	3 (2, 4)	0	7	4 (4, 4)	5 (19)
**Short-term adverse effect**	17	3 (2, 3)	0	17	4 (3, 4)	0

Parents and clinicians generally agreed on which outcomes were most important. However, parents, of both preschool and school-aged children, scored long-term outcomes more highly than did clinicians. Both parents and clinicians considered long-term adverse effects of therapy more important than short-term adverse effects.

## Discussion

### Main findings

We found that the outcomes considered most important in childhood asthma by clinicians, patients, and parents were symptoms, exacerbations, quality of life, and mortality. We identified these by asking clinicians, parents, and young people, what outcomes they reviewed or reported during routine clinical consultations to enable them to decide, jointly, whether or not therapy should be continued, augmented, or reduced. This differs from the approach generally taken in deciding outcomes for clinical trials, which is to focus on what has been measured before [9]. In this pilot study, we have shown that it is feasible, and useful, to involve clinicians, patients, and families, in identifying which outcomes are important when making shared decisions about management in clinical practice.

The frequency and severity of symptoms and exacerbations are important for clinicians and trialists in deciding whether asthma is well controlled [10], so it is unsurprising that they emerged as important components of a core outcome set in this area. Although lung function is frequently assessed in asthma clinical trials because it is an objective evaluation of efficacy, we found that parents and clinicians place much more emphasis on clinical measures of asthma control when assessing the effectiveness of therapy.

We found that, in all age groups, the effects of interventions on measures of functional status, such as the ability to perform normal activities, play sports, and attend school, and also on overall QoL, were fundamental to the assessment of their benefit. Clinical markers of disease, which do not evaluate psychosocial and functional effects of having asthma [10], should not be used as substitutes for such assessments [11]. Although QoL outcomes are assessed relatively infrequently in clinical trials in asthma [4], we recommend that they be included in a core outcome set, either as individual components of functional status or emotional well-being, or as part of a validated composite outcome.

We found that parents place more emphasis on long-term beneficial effects of therapy than clinicians do. Previous qualitative research has identified that many parents of children with asthma worry about long-term effects on health [12], and cohort studies suggest that children with asthma are likely to continue to have asthma as adults [13]. Long-term harmful effects of therapies were important to both clinicians and parents in our study. These have also been shown to be a major concern for parents and children [14], and are an important research priority [15]. We feel that, despite difficulties of measuring long-term effects of therapies, they should be also be part of a core outcome set, although further work is needed to identify the most appropriate ways to measure and report these outcomes. Around half of the parents who participated in phase 2 selected ‘prevention of death’ as one of their top three outcomes, compared with a quarter of clinicians. One reason for this discrepancy may be that clinicians may feel that because death from asthma in childhood is very rare, it should not be part of a core outcome set. We recommend, however, that death should always be reported as a secondary outcome in clinical trials in children with asthma.

### Comparison with the ATS/ERS outcomes taskforce

As mentioned earlier, a recent ATS/ERS taskforce [3] has recommended outcomes that should be measured in clinical trials of regular therapies in asthma. Outcomes measured in clinical trials were identified by a literature review. Working groups comprising clinicians, researchers, and pharmaceutical industry representatives, reached consensus using round-table open discussions about the suitability of these outcomes for evaluating current and future asthma-related problems. Two paediatricians assessed whether the recommendations were applicable to clinical trials in children.

The recommended outcomes, which were symptoms, use of reliever inhaler, composite scores for assessing asthma control, exacerbations, quality of life, pre- and post-bronchodilator FEV1 (for assessment of lung function decline), and adverse effects of therapy, are comparable with our results. The main difference was that participants in our study considered physiological measures of lung function, although ‘essential’ outcomes in the ATS/ERS core set, one of the least important outcomes. Other groups have also highlighted issues relating to whether or not outcomes measured in clinical trials in adults are applicable to children [16]. The discrepancy may also reflect our empirical approach of considering the importance of outcomes in the context of factors that affect decisions in a clinic consultation compared to the ATS/ERS approach of starting with what has been measured previously and assessing the relevance of those outcomes. We acknowledge that FEV1 will continue to be measured in clinical trials in asthma, because it is reliable and repeatable, but our work highlights that other outcomes are of more importance when making clinical decisions.

### Limitations of this study

Our sample focussed on children whose asthma is managed by hospital paediatricians, although much asthma is treated in primary care. The study should be replicated amongst primary care doctors, practice nurses, parents with children whose asthma is managed in primary care, and young people who are treated in this setting. This will also address the fact that only small numbers of young people were included in our study in phase 1. We feel, however, that our results are relevant to a high proportion of clinical trials, which are usually conducted in the hospital setting [17-19]. We have also sampled participants from the UK, and it is possible that if this study were replicated in other countries, some other outcomes may have been found to be of particular importance. For this reason, we feel that future work to develop a more definitive core outcome set for childhood asthma, which is broadly applicable internationally, should incorporate the views of participants from several countries.

Despite good agreement among reviewers, certain responses in phase 1 were open to interpretation. One example related to the description of symptoms. Although some parents specified daytime or nocturnal symptoms, others did not. A pragmatic decision was taken that a description of symptoms should be classified as referring to nocturnal symptoms only if the response was this specific. If our assumption had been false, the number of participants suggesting ‘night-ime symptoms’ could have been underestimated in phase 1, or those suggesting ‘daytime symptoms’ could have been overestimated. Ultimately, our results appear to be robust to this decision, because nocturnal symptoms were included in the phase 2 questionnaire, and daytime symptoms scored highly.

Responses from parents in phase 1 that were classified as representing QoL were also open to interpretation. However, even if we had misinterpreted some responses from parents, in phase 1, this is unlikely to have affected the phase 2 questionnaire. We kept a broad definition of QoL that reflects a patient’s overall well-being, including psychosocial status. A detailed examination of what participants meant by QoL was outside the scope of this study.

Our sample of clinicians represented only 18 % of people to whom the invitation was sent, although our sample size is comparable to other studies that have attempted to determine which outcomes to measure in clinical trials [9]. It is possible that this may have resulted in bias towards participants with specific interest in this type of study. Although we cannot be certain about the views of the 214 non-responders, we see no reason why this group should differ from those who did respond. We assumed that these clinicians did not want to be involved in the study, or were too busy, so we felt it would be inappropriate to send them an email inviting them to phase 2.

## Conclusions

In this study, we have developed and piloted a method for involving patients, parents, and clinicians in the process of identifying relevant outcomes for clinical trials. The replication of this study in other settings will be important for the development of a core outcome set for childhood asthma. We recommend that others adopt this broad empirical approach of attempting to align outcomes for assessing interventions in clinical trials with those that are used for making decisions in clinical practice.

When making shared clinical decisions about daily management of childhood asthma, parents and clinicians wish to know whether an intervention controls symptoms, prevents morbidity and mortality associated with exacerbations, and improves QoL. Outcomes in clinical trials, which generally measure short-term clinical and physiological efficacy, may not be sufficient to fully evaluate the effects of interventions that are meaningful in clinical practice.

A core outcome set for childhood asthma that not only suits the needs of researchers, but also improves the usefulness of clinical trials from the perspective of clinicians, parents, and policy-makers can be based on our findings in conjunction with the recommendations of the recent ATS/ERS taskforce. Agreement amongst a wider group of people involved in such trials should focus on identifying the best ways to measure symptoms and the effects of asthma on daily life. Standardising the definitions of endpoints, especially exacerbations, would also reduce heterogeneity between studies. Further discussion should also address the role of physiological measures of lung function in clinical trials in children with asthma, and whether outcomes reflecting long-term beneficial and harmful effects of treatments should be measured in all such trials.

## Abbreviations

AHCH, Alder Hey Children’s Hospital; ATS, American Thoracic Society; BPRS, British Paediatric Respiratory Society; ERS, European Respiratory Society; FEV1, forced expiratory volume in 1 s; QoL, quality of life; RCT, randomised controlled trial.

## Competing interests

The authors declare that they have no competing interests.

## Author contributions

IS, RLS, and PRW conceived and designed the study. IS collected data, and IS, RG, RLS, and PRW were involved in data analysis. All authors read and approved the final manuscript.

## Financial disclosure

Ian Sinha was funded by the NIHR Medicines for Children Research Network Clinical Trials Unit and Co-ordinating Centre. The Medicines for Children Research Network is part of the National Institute for Health Research (NIHR) and is funded by the Department of Health. The funders had no role in the study design, data collection and analysis, decision to publish, or preparation of the manuscript. The study was funded by Department of Health grant RNC/013/011.

## Supplementary Material

Additional file 1 List of clinicians who participated in the Delphi process (shown with consent from each participant). Click here for file

Additional file 2 Questionnaire used in phase 2. Click here for file

Additional file 3 Results of phase 1. Click here for file

Additional file 4 Summary of agreement between reviewers. Click here for file
