# Supplementary material for: Development of a core outcome set for clinical trials in childhood asthma: a survey of clinicians, parents, and young people
Source: Trials. 2012 Jul 2;13:103. doi: 10.1186/1745-6215-13-103 (PMC3433381; doi:10.1186/1745-6215-13-103)
Supplement: Additional file 1 — List of clinicians who participated in the Delphi process (shown with consent from each participant). [file 1745-6215-13-103-S1.pdf]

Additional File 1 - List of clinicians who participated in Round 1 of the Delphi process

Name	Job title	Institution
Ian Balfour-Lynn	Consultant respiratory paediatrician	Royal Brompton Hospital
Jayesh M Bhatt	Consultant respiratory paediatrician	Nottingham University Hospitals
Cara Bossley	Clinical Research Fellow	Royal Brompton Hospital
Hannah Buckley	Consultant paediatrician	Portsmouth Hospitals NHS Trust
Fran Child	Consultant respiratory paediatrician	Royal Manchester Childrens' Hospital
Jayne Clarke	Consultant paediatrician with respiratory interest	Hereford County Hospital
Sadie Clayton	Senior paediatric respiratory nurse	University Hospital North Staffordshire
Gary Connett	Consultant paediatrician	Southampton University Hospitals Trust
Donna Corrigan	Consultant paediatrician with respiratory interest	Wishaw General Hospital
Jon Couriel	Consultant respiratory paediatrician	Alder Hey Children's Hospital
Margaret Cuffwright	Specialist health visitor – paediatric asthma	Salford PCT/Central Manchester Foundation trust
Suparna Dasgupta	Consultant paediatrician	North Manchester General Hospital
Anne Devenney	Consultant respiratory paediatrician	Royal Hospital for Sick Children (Yorkhill), Glasgow
Amanda Equi	Consultant paediatrician with respiratory interest	West Hertfordshire NHS Trust
Hazel Evans	Consultant respiratory paediatrician	Childrens Hospital for Wales
Debra Forster	Childrens' respiratory nurse	Nottingham University Hospitals
John Furness	Consultant paediatrician	County Durham & Darlington NHS Foundation Trust
Jonathan Garside	Consultant paediatrician	Calderdale and Huddersfield NHS Foundation Trust
Neil Gibson	Consultant respiratory paediatrician	Royal Hospital for Sick Children (Yorkhill), Glasgow
Charles Godden	Consultant paediatrician	Royal Surrey County Hospital
Ajay Gupta	Consultant paediatrician	Stafford General Hospital
Peter J Helms	Professor of Child Health	University of Aberdeen
Sheila Javadpour	Consultant respiratory paediatrician	Alder Hey Children's Hospital, Liverpool
Helen Jones	Specialist Nurse	St George's NHS Trust

Tim Lee	Consultant respiratory paediatrician	St James' Hospital, Leeds
Warren Lenney	Professor of Respiratory Child Health and Consultant Respiratory Paediatrician	Keele University and University Hospital of North Staffordshire
BG Loftus	Professor of paediatrics	NUI Galway
Anirban Maitra	Specialist Registrar in Respiratory Paediatrics	Sheffield Children's Hospital
Melanie McFeeters	Consultant nurse	University Hospitals Leicester NHS Trust
Godfrey Nyamugunduru	Consultant paediatrician	University Hospital of North Durham
Sheila Peters	Consultant paediatrician	Portsmouth Hospitals NHS Trust
Ian Pollock	Consultant paediatrician	Chase Farm Hospital
Robert Primhak	Consultant respiratory paediatrician	Sheffield Children's Hospital
Rosemary Rayner	Consultant paediatrician	New Cross Hospital, Wolverhampton
Robert Ross Russell	Consultant in PICU and Respiratory Paediatrics	Addenbrooke's Hospital, Cambridge
Martin Samuels	Consultant paediatrician	University Hospital North Staffordshire
MG Semple	Senior Lecture and Consultant in Paediatric Respiratory Medicine	Alder Hey Children's Hospital
Michael D Shields	Professor of Child Health	Royal Belfast Hospital for Sick Children
David Spencer	Consultant respiratory paediatrician	Newcastle Hospitals
Anne Thomson	Consultant respiratory paediatrician	Oxford Childrens' Hospital
Steve Turner	Senior lecturer	University of Aberdeen
Chris Upton	Consultant paediatrician	Norfolk & Norwich University Hospital
Lynette Williams	Paediatric respiratory nurse specialist	Shrewsbury and Telford NHS trust
Nicola Wilson	Honorary consultant in respiratory paediatrics	Royal Brompton Hospital
Edwina Wooler	Senior paediatric respiratory nurse specialist	Royal Alexandra Children's Hospital, Brighton
Caroline Youle	Respiratory nurse specialist	Nottingham University Hospital
