# Supplementary material for: Development of a core outcome set for clinical trials in childhood asthma: a survey of clinicians, parents, and young people
Source: Trials. 2012 Jul 2;13:103. doi: 10.1186/1745-6215-13-103 (PMC3433381; doi:10.1186/1745-6215-13-103)
Supplement: Additional file 2 — Questionnaire used in phase 2. [file 1745-6215-13-103-S2.pdf]

Additional file 2 – Questionnaires distributed to clinicians and parents in Phase 2

ASTHMA OUTCOMES - SURVEY OF HEALTH PROFESSIONALS

Thank you for participating in the second part of our study

Before completing the questionnaire, please read this important background information:

Clinical trials are research studies. They are conducted to determine which treatments are best for patients with a given condition.

In clinical trials we aim to measure various effects of treatments, both beneficial and harmful, over a period of time. We refer to this as measuring the effects of treatments on various outcomes.

We would like to know which outcomes you feel are important, and should be measured in clinical trials of regular preventer treatments for children with asthma.

We will be asking you to consider outcomes in relation to pre-school and school age children separately

Please tell us your FULL NAME*

Regular treatments for children can have a variety of beneficial effects, each of which could be measured as an outcome in clinical trials.

Please score how important each of the following outcomes are on a scale of 0-4 in relation to SCHOOL-AGE CHILDREN (5 Years or older)

0= not at all important
(you are not concerned if this is measured in clinical trials or not)

4= very important
(you feel this is important and should be measured in clinical trials)

1. The effect of a treatment on daytime asthma symptoms

(eg cough, wheeze, shortness of breath)
○ 0 ○ 1 ○ 2 ○ 3 ○ 4

2. The effect of a treatment on night-time asthma symptoms

(eg coughing during sleep)
○ 0 ○ 1 ○ 2 ○ 3 ○ 4

3. The effect of a treatment on activity or exercise without asthma symptoms
○ 0 ○ 1 ○ 2 ○ 3 ○ 4

4. The effect of a treatment on how often children need to use their reliever inhaler
○ 0 ○ 1 ○ 2 ○ 3 ○ 4

5. The effect of a treatment on how often children have exacerbations of asthma that require a course of oral steroids (prednisolone)
○ 0 ○ 1 ○ 2 ○ 3 ○ 4

6. The effect of a treatment on a child's overall asthma control (as judged by the parents and the child, rather than by doctors)
○ 0 ○ 1 ○ 2 ○ 3 ○ 4

7. The effect of a treatment on lung function tests

○ 0 ○ 1 ○ 2 ○ 3 ○ 4

8. The effect of a treatment on preventing asthma-related death

○ 0 ○ 1 ○ 2 ○ 3 ○ 4

9. The effect of a treatment on how well children can do normal activities that they would like to do (eg playing sport, or socialising with friends)

○ 0 ○ 1 ○ 2 ○ 3 ○ 4

10. The effect of a treatment on the amount of school a child misses because of asthma

○ 0 ○ 1 ○ 2 ○ 3 ○ 4

11. The effect of a treatment on the overall quality of life of children with asthma

○ 0 ○ 1 ○ 2 ○ 3 ○ 4

12. The effect of a treatment on how frequently children visit a GP or A+E department because of asthma

○ 0 ○ 1 ○ 2 ○ 3 ○ 4

13. The effect of a treatment on how frequently children need to be admitted to hospital because of asthma

○ 0 ○ 1 ○ 2 ○ 3 ○ 4

14. The effect of a treatment on health-related problems when children are older

○ 0 ○ 1 ○ 2 ○ 3 ○ 4

Please score how important you think the following side effects are for SCHOOL AGE CHILDREN

15. Short term side effects that stop when the treatment stops

○ 0 ○ 1 ○ 2 ○ 3 ○ 4

16. Effects of treatment on growth

○ 0 ○ 1 ○ 2 ○ 3 ○ 4

17. Other long-term side effects that continue, or appear, after the treatment stops

○ 0 ○ 1 ○ 2 ○ 3 ○ 4

18. You have just scored the outcomes listed below.

Please tick the THREE MOST IMPORTANT in relation to SCHOOL-AGE children*

- ☐ The effect of a treatment on daytime symptoms
- ☐ The effect of a treatment on night-time symptoms
- ☐ The effect of a treatment on activity or exercise without symptoms
- ☐ The effect of a treatment on how often children need to use their reliever inhaler
- ☐ The effect of a treatment on exacerbations of asthma that require oral steroids
- ☐ The effect of a treatment on overall asthma control (judged by parent and child)
- ☐ The effect of a treatment on lung function tests
- ☐ The effect of a treatment on preventing asthma-related death
- ☐ The effect of treatment on how well a child can do normal activities
- ☐ The effect of a treatment on school missed because of asthma
- ☐ The effect of a treatment on a child's overall quality of life
- ☐ The effect of a treatment on visits to a GP or A+E department
- ☐ The effect of a treatment on admissions to hospital
- ☐ The effect of a treatment on health-related problems when a child is older
- ☐ Short term side effects when the treatment stops
- ☐ Effects of treatment on growth
- ☐ Other long-term side effects that continue or appear after the treatment stops

19. Are there any other outcomes of treatment which are not listed that would have made it into your top 3?

Thank you

Now please score how important each of the following outcomes are on a scale of 0-4 in relation to PRE- SCHOOL CHILDREN (younger than 5 years)

0= not at all important
(you are not concerned if this is measured in clinical trials or not)

4= very important
(you feel this is important and should be measured in clinical trials)

20. The effect of a treatment on daytime asthma symptoms

(eg cough, wheeze, shortness of breath)

○ 0 ○ 1 ○ 2 ○ 3 ○ 4

21. The effect of a treatment on night-time asthma symptoms

(eg coughing during sleep)

○ 0 ○ 1 ○ 2 ○ 3 ○ 4

22. The effect of a treatment on activity or exercise without asthma symptoms

○ 0 ○ 1 ○ 2 ○ 3 ○ 4

23. The effect of a treatment on how often children need to use their reliever inhaler

○ 0 ○ 1 ○ 2 ○ 3 ○ 4

24. The effect of a treatment on how often children have exacerbations of asthma that require a course of oral steroids (prednisolone)

○ 0 ○ 1 ○ 2 ○ 3 ○ 4

25. The effect of a treatment on a child's overall asthma control (as judged by the parents and the child, rather than by doctors)

○ 0 ○ 1 ○ 2 ○ 3 ○ 4

26. The effect of a treatment on preventing asthma-related death

○ 0 ○ 1 ○ 2 ○ 3 ○ 4

27. The effect of a treatment on how well children can do normal activities that they would like to do (eg playing sport, or socialising with friends)

○ 0 ○ 1 ○ 2 ○ 3 ○ 4

28. The effect of a treatment on the amount of school/nursery a child misses because of asthma

○ 0 ○ 1 ○ 2 ○ 3 ○ 4

29. The effect of a treatment on the overall quality of life of children with asthma

○ 0 ○ 1 ○ 2 ○ 3 ○ 4

30. The effect of a treatment on the impact of asthma on the whole family (eg number of days of work that parents miss because of a child's asthma, or parental anxiety)

○ 0 ○ 1 ○ 2 ○ 3 ○ 4

31. The effect of a treatment on how frequently children visit a GP or A+E department because of asthma

○ 0 ○ 1 ○ 2 ○ 3 ○ 4

32. The effect of a treatment on how frequently children need to be admitted to hospital because of asthma

○ 0 ○ 1 ○ 2 ○ 3 ○ 4

33. The effect of a treatment on health-related problems when children are older

○ 0 ○ 1 ○ 2 ○ 3 ○ 4

Please score how important you think the following side effects are for PRE-SCHOOL CHILDREN

34. Short term side effects that stop when the treatment stops

○ 0 ○ 1 ○ 2 ○ 3 ○ 4

35. Effects of treatment on growth

○ 0 ○ 1 ○ 2 ○ 3 ○ 4

36. Other long-term side effects that continue, or appear, after the treatment stops

○ 0 ○ 1 ○ 2 ○ 3 ○ 4

37. **You have just scored the outcomes listed below.**

Please tick the THREE MOST IMPORTANT in relation to PRE-SCHOOL children*

- ☐ The effect of a treatment on daytime symptoms
- ☐ The effect of a treatment on night-time symptoms
- ☐ The effect of a treatment on activity or exercise without symptoms
- ☐ The effect of a treatment on how often children need to use their reliever inhaler
- ☐ The effect of a treatment on exacerbations of asthma that require oral steroids
- ☐ The effect of a treatment on overall asthma control (judged by parent and child)
- ☐ The effect of a treatment on preventing asthma-related death
- ☐ The effect of treatment on how well a child can do normal activities
- ☐ The effect of a treatment on school/nursery missed because of asthma
- ☐ The effect of a treatment on overall quality of life
- ☐ The effect of a treatment on the overall impact of asthma on the family
- ☐ The effect of a treatment on visits to a GP or A+E department
- ☐ The effect of a treatment on admissions to hospital
- ☐ The effect of a treatment on health-related problems when a child is older
- ☐ Short term side effects when the treatment stops
- ☐ Effects of treatment on growth
- ☐ Other long-term side effects that continue or appear after the treatment stops

38. **Are there any other outcomes of treatment which are not listed that would have made it into your top 3?**

Finished? Submit your Survey

Medicines for Children Research Network

ASTHMA OUTCOMES - SURVEY OF PARENTS

Dear Parent

We are very grateful that you have agreed to participate in our study.

Before completing the questionnaire, please read this important background information:

Clinical trials are research studies. They are conducted to determine which treatments are best for patients with a given condition.

In clinical trials we aim to measure various effects of treatments, both beneficial and harmful, over a period of time. We refer to this as measuring the effects of treatments on various outcomes.

We would like to know which outcomes you, as a parent, feel are important, and should be measured in clinical trials of regular preventer treatments for children with asthma.

How old is your child, in years?*

-- Please Select --

Regular treatments for children can have a variety of beneficial effects, each of which could be measured as an outcome in clinical trials.

Please score how important each of the following outcomes are on a scale of 0-4

0= not at all important
(you are not concerned if this is measured in clinical trials or not)

4= very important
(you feel this is important and should be measured in clinical trials)

1. **The effect of a treatment on daytime asthma symptoms**

(eg cough, wheeze, shortness of breath)

○ 0 ○ 1 ○ 2 ○ 3 ○ 4

2. **The effect of a treatment on night-time asthma symptoms**

(eg coughing during sleep)

○ 0 ○ 1 ○ 2 ○ 3 ○ 4

3. **The effect of a treatment on activity or exercise without asthma symptoms**

○ 0 ○ 1 ○ 2 ○ 3 ○ 4

4. The effect of a treatment on how often children need to use their reliever inhaler

○ 0 ○ 1 ○ 2 ○ 3 ○ 4

5. The effect of a treatment on how often children have exacerbations of asthma that require a course of oral steroids (prednisolone)

○ 0 ○ 1 ○ 2 ○ 3 ○ 4

6. The effect of a treatment on a child's overall asthma control (as judged by the parents and the child, rather than by doctors)

○ 0 ○ 1 ○ 2 ○ 3 ○ 4

7. The effect of a treatment on lung function tests (blowing tests)

[PLEASE SCORE THIS OUTCOME ONLY IF YOUR CHILD IS AGED FIVE YEARS OR OLDER]

○ 0 ○ 1 ○ 2 ○ 3 ○ 4

8. The effect of a treatment on preventing asthma-related death

○ 0 ○ 1 ○ 2 ○ 3 ○ 4

9. The effect of a treatment on how well children can do normal activities that they would like to do (eg playing sport, or socialising with friends)

○ 0 ○ 1 ○ 2 ○ 3 ○ 4

10. The effect of a treatment on the amount of school/nursery a child misses because of asthma

○ 0 ○ 1 ○ 2 ○ 3 ○ 4

11. The effect of a treatment on the overall quality of life of children with asthma

○ 0 ○ 1 ○ 2 ○ 3 ○ 4

12. The effect of a treatment on the impact of asthma on the whole family (eg number of days of work parents miss because their child's asthma is bad)

[PLEASE SCORE THE OUTCOME 'IMPACT OF ASTHMA ON THE WHOLE FAMILY' ONLY IF YOUR CHILD IS YOUNGER THAN FIVE YEARS OF AGE]

○ 0 ○ 1 ○ 2 ○ 3 ○ 4

13. The effect of a treatment on how frequently children visit a GP or A+E department because of asthma

○ 0 ○ 1 ○ 2 ○ 3 ○ 4

14. The effect of a treatment on how frequently children need to be admitted to hospital because of asthma

○ 0 ○ 1 ○ 2 ○ 3 ○ 4

15. The effect of a treatment on health-related problems when children are older

○ 0 ○ 1 ○ 2 ○ 3 ○ 4

Treatments for children with asthma can also have side-effects.

Please score how important you think the following side effects are, using the same scale of 0-4

16. Short term side effects that stop when the treatment stops

○ 0 ○ 1 ○ 2 ○ 3 ○ 4

17. Effects of treatment on growth

○ 0 ○ 1 ○ 2 ○ 3 ○ 4

18. Other long-term side effects that continue, or appear, after the treatment stops

○ 0 ○ 1 ○ 2 ○ 3 ○ 4

19. You have just scored the outcomes listed below.

Please tick the THREE MOST IMPORTANT outcomes*

- ☐ The effect of a treatment on daytime symptoms
- ☐ The effect of a treatment on night-time symptoms
- ☐ The effect of a treatment on activity or exercise without symptoms
- ☐ The effect of a treatment on how often children need to use their reliever inhaler
- ☐ The effect of a treatment on exacerbations of asthma that require oral steroids
- ☐ The effect of a treatment on overall asthma control (judged by parent and child)
- ☐ The effect of treatment on lung function (blowing) tests
- ☐ The effect of a treatment on preventing asthma-related death
- ☐ The effect of treatment on how well a child can do normal activities
- ☐ The effect of a treatment on school/nursery missed because of asthma
- ☐ The effect of a treatment on overall quality of life
- ☐ The effect of a treatment on the impact of asthma on the whole family
- ☐ The effect of a treatment on visits to a GP or A+E department
- ☐ The effect of a treatment on admissions to hospital
- ☐ The effect of a treatment on health-related problems when a child is older
- ☐ Short term side effects when the treatment stops
- ☐ Effects of treatment on growth
- ☐ Other long-term side effects that continue or appear after the treatment stops

20. Are there any other outcomes of treatment which are not listed that would have made it into your top 3?

[ ]
