# Supplementary material for: Development of a core outcome set for clinical trials in childhood asthma: a survey of clinicians, parents, and young people
Source: Trials. 2012 Jul 2;13:103. doi: 10.1186/1745-6215-13-103 (PMC3433381; doi:10.1186/1745-6215-13-103)
Supplement: Additional file 3 — Results of phase 1. [file 1745-6215-13-103-S3.doc]

|  |  |  |
| --- | --- | --- |
|  |  |  |
|  |  |  |
|  |  |  |
|  |  |  |
|  |  |  |
|  |  |  |
|  |  |  |
|  |  |  |
|  |  |  |
|  |  |  |
|  |  |  |
|  |  |  |
|  |  |  |
|  |  |  |
|  |  |  |
|  |  |  |
|  |  |  |
|  |  |  |
|  |  |  |
|  |  |  |
|  |  |  |

|  |  |  |
| --- | --- | --- |
|  |  |  |
|  |  |  |
|  |  |  |
|  |  |  |
|  |  |  |
|  |  |  |
|  |  |  |
|  |  |  |
|  |  |  |
|  |  |  |
|  |  |  |
|  |  |  |
|  |  |  |
|  |  |  |
|  |  |  |
|  |  |  |
|  |  |  |
|  |  |  |
|  |  |  |
|  |  |  |
|  |  |  |

Footnotes:

a: both clinicians suggested Exhaled Nitric Oxide (ENO)

|  |  |  |
| --- | --- | --- |
|  |  |  |
|  |  |  |
|  |  |  |
|  |  |  |
|  |  |  |
|  |  |  |
|  |  |  |
|  |  |  |
|  |  |  |
|  |  |  |
|  |  |  |
|  |  |  |
|  |  |  |
|  |  |  |
|  |  |  |
|  |  |  |
|  |  |  |
|  |  |  |

|  |  |  |
| --- | --- | --- |
|  |  |  |
|  |  |  |
|  |  |  |
|  |  |  |
|  |  |  |
|  |  |  |
|  |  |  |
|  |  |  |
|  |  |  |
|  |  |  |
|  |  |  |
|  |  |  |
|  |  |  |
|  |  |  |
|  |  |  |
|  |  |  |
|  |  |  |
|  |  |  |
|  |  |  |

|  |  |  |
| --- | --- | --- |
|  |  |  |
|  |  |  |
|  |  |  |
|  |  |  |
|  |  |  |
|  |  |  |
|  |  |  |
|  |  |  |
|  |  |  |
|  |  |  |
|  |  |  |
|  |  |  |
|  |  |  |

**Additional file 3 Results from the Phase 1 questionnaires**

**: Whether outcomes were carried forward to Phase 2, including the frequency with which they were suggested by participants, and measured in RCTs in childhood asthma**

| **Outcome** | **Clinicians School age children (n=46)** | **Clinicians**  **Pre school children (n=46)** | **Parents of school age children (n=27)** | **Young people (n=11)** | **Parents of preschool children (n=11)** | **RCTs including school age children (n=95)** | **RCTs including pre-school children (n=47)** | **Is outcome carried forward for school age children** | **Is outcome carried forward for pre-school age children** | **Notes** |
| --- | --- | --- | --- | --- | --- | --- | --- | --- | --- | --- |
| **DISEASE ACTIVITY** |  |  |  |  |  |  |  |  |  |  |
| **Daytime symptoms** | 43 (92) | 43 (93) | 9 (33) | 5 (45) | 8 (73) | 84 (88) | 43 (91) | Yes | Yes | 84/95 and 43/47 RCTs measured symptoms. Daytime, nocturnal and exercise-induced symptoms were included in these scores. |
| **Nocturnal symptoms** | 30 (63) | 28 (61) | 20 (74) | 2 (18) | 1 (9) |  |  | Yes | Yes |  |
| **Symptoms affecting activity (eg exercise)** | 29 (63) | 17 (37) | 11 (41) | 4 (36) | 3 (27) |  |  | Yes | Yes |  |
| **Need for bronchodilator** | 27 (58) | 23 (50) | 3 (11) | 0 | 2 (18) | 71 (75) | 39 (83) | Yes | Yes |  |
| **Exacerbations** | 31 (67) | 29 (63) | 13 (48) | 1 (9) | 4 (36) | 31 (33) | 17 (36) | Yes | Yes | This outcome includes suggestions by clinicians, that exacerbations, specifically in response to URTIs, are an important outcome |
| **Patient/parent overall view on asthma control** | 3 (6) | 3 (6) | 5 (19) | 0 | 1 (9) | 9 (9) | 6 (13) | Yes | Yes | Although parents did not specifically describe this outcome, 5 parents did describe terms relating to overall asthma control |
| **Lung function** | 14 (30) | 0 | 1 (4) | 0 | 0 | 89 (94) | 36 (77) | Yes | No | Not carried forward for pre-school children because it was suggested by no parents or clinicians |
| **Bronchoconstriction induced by challenge** | 0 | 0 | 0 | 0 | 0 | 28 (30) | 1 (2) | No | No | Not carried forward for school-age children because it was suggested by no parents or clinicians |
| **Inflammatory markers** | 2 (4) | 0 | 0 | 0 | 0 | 16 (17) | 3 (6) | No | No | Not carried forward (said by 2/46 clinicians; no parents) |
| **Ability to feed** | 0 | 2 (4) | 1 (4) | 0 | 1 (9) | * | * | No | No | *May have been measured as part of symptom score in RCTs, but not as an outcome in itself |
| **Occurrence of wheeze in response to allergens** | 0 | 3 (7) | 0 | 0 | 0 | * | * | No | No | *May have been measured as part of symptom score in RCTs, but not as an outcome in itself |
| **Normal chest exam** | 2 (4) | 3 (7) | 0 | 0 | 0 | 0 | 0 | No | No |  |
| **Change in FEV1 in response to B2+** | 2 (4) | 3 (7) | 0 | 0 | 0 | 0 | 0 | No | No |  |
| **Outcome** | **Clinicians School age children (n=46)** | **Clinicians**  **Pre school children (n=46)** | **Parents of school age children (n=27)** | **Young people (n=11)** | **Parents of preschool children (n=11)** | **RCTs including school age children** | **RCTs including pre-school children** | **Included for school age children** | **Included for pre-school age children** | **Notes** |
| **DISEASE DAMAGE** |  |  |  |  |  |  |  |  |  |  |
| **Death** | 0 | 1 (2) | 3 (11) | 1 (9) | 0 | * | * | Yes | Yes | *Measured implicitly but not reported |
| **Lung growth** | 0 | 0 | 0 | 0 | 0 | 1 (1) | 0 | No | No |  |
| **Development milestones** | 1 (2) | 0 | 0 | 0 | 0 | 0 | 0 | No | No |  |
| **FUNCTIONAL STATUS** |  |  |  |  |  |  |  |  |  |  |
| **ADL** | 14 (30) | 9 (20) | 13 (48) | 3 (27) | 2 (18) | 6 (6) | 5 (10) | Yes | Yes |  |
| **School/nursery** | 27 (59) | 5 (11) | 2 (7) | 0 | 0 | 13 (14) | 4 (4) | Yes | Yes |  |
| **QoL/ FAMILY OUTCOMES** |  |  |  |  |  |  |  |  |  |  |
| **Overall QoL** | 7 (15) | 4 (9) | 4 (15) | 1 (9) | 1 (9) | 7 (7) | 5 (11) | Yes | Yes |  |
| **Emotional well being** | 0 | 0 | 1 (4) | 1 (9) | 1 (9) | * | * | No | No | Incorporated in QoL scores, but not measured as an outcome itself |
| **Family outcomes** | 1 (2) | 8 (17) | 0 | 0 | 0 | 7 (7) | 10 (21) | No | Yes |  |
| **ADVERSE EFFECTS OF THERAPY** |  |  |  |  |  |  |  |  |  |  |
| **Short term AE** | 6 (13) | 10 (22) | 16 (60) | 2 (18) | 5 (45) | 78 (82)* | 43 (96) * | Yes | Yes | * Did not distinguish between short and long term AEs |
| **Growth** | 11 (24) | 8 (17) | 2 (7) | 1 (9) | 1 (9) | 30 (32) | 16 (34) | Yes | Yes |  |
| **Other long term AE** | 15 (30) | 1 (2) | 14 (52) | 3 (27) | 4 (36) | * | * | Yes | Yes | * Did not distinguish between short and long term AEs |
| **Dependence** | 0 | 0 | 2 (7) | 0 | 1 (9) | 0 | 0 | No | No |  |
| **HEALTH RESOURCE UTILISATION** |  |  |  |  |  |  |  |  |  |  |
| **A+E/GP visit** | 7 (15) | 9 (18) | 2 (7) | 0 | 0 | 8 (8) | 7 (15) | Yes | Yes | * Either included in exacerbations or unscheduled HRU |
| **Hospital admission** | 13 (28) | 20 (45) | 2 (7) | 1 (9) | 0 | * | * | Yes | Yes | * Either included in exacerbations or unscheduled HRU |
| **OTHER** |  |  |  |  |  |  |  |  |  |  |
| **Health-related problems when child is older** | 0 | 0 | 7 (26) | 1 (9) | 2 (18) | 0 | 0 | Yes | Yes | Includes disease damage (ie progression of respiratory illness into older childhood or adulthood) and long term functional status (eg career, lifestyle when older) |
| **Achieving parent/ patient-defined goal** | 1 (2) | 0 | 0 | 0 | 0 | 0 | 0 | No | No |  |

*: Issues relating to the reporting of outcomes in RCTs are described in the column entitled ‘notes’

Abbreviations: ADL=Activities of Daily Living; AE=Adverse effect; A+E=Accident and Emergency; B2+= Beta agonist; FEV1= Forced Expiratory volume in 1 second; ICS= Inhaled corticosteroids; PEFR=Peak Expiratory Flow Rate; QoL=Quality of life; URTI=upper respiratory tract infection
