# Supplementary material for: Development of a core outcome set for clinical trials in childhood asthma: a survey of clinicians, parents, and young people
Source: Trials. 2012 Jul 2;13:103. doi: 10.1186/1745-6215-13-103 (PMC3433381; doi:10.1186/1745-6215-13-103)
Supplement: Additional file 4 — Summary of agreement between reviewers. [file 1745-6215-13-103-S4.pdf]

Additional file 4 – summary of agreement between reviewers

Agreement between reviewers assessing the Phase 1 questionnaires

Interpretation of answers from clinicians (IS and reviewer 3 (PRW))

Reviewer 3, who was not aware of IS's interpretation of the individual questionnaires, was shown a randomly selected sample of 9/46 clinician questionnaires (20%), and found complete agreement with this analysis.

Interpretation of answers from parents and young people: independent analysis by IS and reviewer 2 (RG)

Reviewer 2 was shown a sample of 36 completed questionnaires, from 27 parents and 9 young people. Reviewer 1 felt that, from the responses in these 36 questionnaires, participants had suggested a total of 124 items that could be interpreted as representing an outcome, and Reviewer 2 felt they had suggested 102. Of the 124 items that Reviewer 1 had extracted, Reviewer 2 agreed with 100 (81%). In total, there were 26 disagreements, of which 24/26 occurred when Reviewer 1 had categorised a response as an outcome but Reviewer 2 had not, and 2/26 had occurred when Reviewer 2 had categorised a response as an outcome but reviewer 1 had not.

Arbitration of the disagreements between IS and Reviewer 2: Discussion with Reviewers 3 (PRW) and 4(RLS)

Reviewer 3 assessed all 26 of these disagreements. With regard to 20/26 (77%), she agreed with IS. On the 6/26 occasions (23%) that she agreed with Reviewer 2, IS had

classified a response as an outcome but Reviewer 2 did not. IS discussed each of these six responses with Reviewer 3.

After discussion about one response, IS agreed with Reviewer 3. This related to a parent's description that their child "saw [the] asthma nurse who confirmed that his results through breathing tests are much better". IS initially thought this meant that the parents felt lung function was an important outcome. After discussion with Reviewer 3, IS agreed that the parents were simply reporting what the nurse did, rather than saying lung function is an important outcome.

The other five disagreements were arbitrated by Reviewer 4, who agreed with IS's assessment about four of these. IS had thought the outcome "overall asthma control" was most appropriate for the following responses from parents: "[she] has her asthma well controlled through the medication she takes"; "we do not feel it controls [his] asthma"; "her asthma is more under control"; "well controlled". Reviewer 3 had suggested that the comments were not detailed enough to interpret in this way. Reviewer 4 suggested IS's classification was appropriate, because "overall asthma control" is a concept that parents often discuss in out-patient consultations.

One disagreement related to whether outcomes relating to exercise should be categorised in the functional status domain (ie ability to exercise) or the disease activity domain (ie exercise-related symptoms). After discussion with Reviewer 4, IS agreed that this outcome is more appropriately placed in the disease activity domain.

Discussion of atypical comments between IS and Reviewer 4 (RLS)

Reviewer 4 was asked to check 18 responses, which IS felt may be open to interpretation. IS and Reviewer 2 had agreed on the classification of these responses. Reviewer 4 deemed the classification to be correct on 16/18 occasions. After discussion, IS agreed with Reviewer 4 that the classification should be changed for the other two responses.

For one of these, Reviewer 2 and IS had interpreted the response "the way my child can be normal and then fighting for breath" as relating to an exacerbation of asthma, but

Reviewer 4 suggested this was just a description of acute shortness of breath, a frequent interval symptom which does not, in itself, constitute an exacerbation. For the other, Reviewer 2 and IS had classified "staying on medication for a long period" as representing a worry about having asthma later in life. Reviewer 4 disagreed with this classification, and suggested that this is more likely to represent a concern about long-term complications of asthma therapy.

Interpretation of responses which did not fit exactly into the initial outcomes framework: discussion amongst IS, Reviewer 3 and Reviewer 4

Symptoms

Symptoms were described in a variety of ways. Some parents responses described cough, wheeze, and shortness of breath. These were all classified as "symptoms", rather than listing them separately on the Phase 2 questionnaire. Where parents specified that these were nocturnal problems, they were classed as such. Therefore, symptoms were classed as either nocturnal or daytime. Some parents discussed symptoms as part of a response to upper respiratory tract infections. It was felt that these could represent exacerbations of asthma, and were classed as such.

Risk of problems in the future

Responses relating to long-term respiratory or overall health, and future impact of asthma on children's lives, were felt to be related. It was difficult to determine whether participants were referring to future risk of asthma, other respiratory illness, general health problems, or problems with functional status. It was also difficult to discern whether 'future risk' referred to later childhood or adulthood, so we felt it would be inappropriate to divide these into separate outcomes based on our interpretation of responses. These were, therefore, combined into the outcome 'health related problems when older'. This outcome was not classed into any of the pre-defined domains, as it was felt to span several of them, but rather in a separate 'other' category.

Adverse effects of medication

Adverse effects of medications were grouped together into three outcomes, namely short-term problems, growth, and other long-term problems. Short-term problems included non-systemic adverse effects, and other comments about side-effects, unless they were specified by participants as relating to growth or other long-term problems. Long-term problems included systemic side effects, and responses from participants that described a future risk from medications because of long-term use.

Quality of life

Certain responses from parents were interpreted as referring to overall quality of life, because they either alluded to parents describing a child's overall well being, or discussed a combination of functional and emotional problems from having asthma. IS and Reviewer 2 had complete agreement that these should be categorised as quality of life. Reviewer 4 had seen all these responses, and also agreed that they described overall quality of life. These responses are shown below:

(1) "[She] is able to live her day-to-day life (school and home) as a normal child";

(2) "I worry that as she gets older her asthma gets worse and will prevent her from leading a normal life";

(3) "His general day-to-day quality of life";

(4) "Difficulties in controlling symptoms, problematic breathing having impact on aspects of daily living, sports, socialising with friends and sleep. I am concerned my son has had anxiety due to worrying about asthma"

(5) "I feel happy because I can do more";

(6) "Not as tired during the day and not as bad tempered. Eating habits have greatly improved – generally more engaged in life"

Responses which were not classed as outcomes

Some suggestions were not categorised as outcomes of treatment, because they did not relate to whether a treatment works, or does more good than harm.

Four clinicians and one set of parents suggested that the ease which parents can administer the asthma therapy, and whether they have the correct technique for using inhalers, are important outcomes. We felt that, although these may affect the decision about whether to change a treatment modality, they do not reflect the efficacy or safety of therapy.

Five clinicians suggested compliance with treatment to be an important outcome. We felt this not to be an outcome of treatment, as a variety of factors determine whether parents and young people comply with a therapy regime, not just whether it is felt to be efficacious and safe.

One young person said she was worried about whether, in the future, her children will be at risk of having asthma. We did not consider this to be an outcome associated with asthma treatments.

One clinician suggested that, in clinic appointments with pre-school children, it is important to use licensed medications.
